# Locoregional tumor failure after definitive radiation for patients with stage III non-small cell lung cancer

**DOI:** 10.1186/1748-717X-9-187

**Published:** 2014-08-26

**Authors:** Raj S Rajpara, Eduard Schreibmann, Tim Fox, Liza J Stapleford, Jonathan J Beitler, Walter J Curran, Kristin A Higgins

**Affiliations:** Department of Radiation Oncology, Emory University School of Medicine and Winship Cancer Institute of Emory University, Atlanta, GA 30322 USA

**Keywords:** Lung cancer, Locally advanced, Locoregional failure, PET/CT, Radiation, Chemotherapy

## Abstract

**Background:**

Locoregional tumor failure (LRF) after definitive chemoradiation for patients with stage III NSCLC remains unacceptably high. This analysis sought to further define where LRF occurs relative to radiation dose received and pre-treatment PET scan-defined maximum standard uptake value (SUVmax).

**Methods:**

This was a retrospective study analyzing patients with stage III NSCLC treated with definitive radiation between 2006 and 2011. LRF was defined as failure within the ipsilateral lung, hilum or mediastinum. The CT simulation scan with the radiation dose distribution was registered to the CT or PET/CT documenting LRF. The region of LRF was contoured, and the dose to 95% of the volume (D95) of LRF was extracted. The pre-treatment SUVmax was also extracted for the anatomic region of LRF.

**Results:**

Sixty-one patients were identified. Median follow-up time was 19.1 months (range 2.37-76.33). Seventy four percent of patients were treated with 3-D conformal technique (3DCRT), 15% were treated with Intensity Modulated Radiotherapy (IMRT), and 11% were treated with a combination of 3DCRT and IMRT. Median prescribed radiation dose for all patients was 66 Gy (39.6-74). Concurrent chemotherapy was delivered in 90% of patients. Twenty-two patients (36%) developed a LRF, with a total of 39 anatomic regions of LRF identified. Median time to LRF was 11.4 months (3.5-44.6). Failures were distributed as follows: 36% were in-field failures, 27% were out-of-field failures, 18% were in-field and out-of-field failures, and 18% were in-field and marginal (recurrences within the field edge) failures. There were no isolated marginal failures. Of the patients that developed a LRF, 73% developed a LRF with an in-field component. Sixty-two percent of LRFs were nodal. The median pre-treatment SUVmax for the anatomic region of LRF for patients with an in-field failure was 13. The median D95 of in-field LRF was 63 Gy.

**Conclusions:**

LRF after definitive chemoradiation are comprised primarily of in-field failures, though out-of field failures are not insignificant. Marginal failures are rare, indicating field margins are appropriate. Although radiation dose escalation to standard radiation fields has not yielded success, using PET parameters to define high-risk regions remains worthy of further investigation.

## Background

Lung cancer continues to be the leading cause of cancer death in the United States [1]. In 2013, an estimated 228,000 new cases of lung cancer were diagnosed and 160,000 lung cancer-related deaths were estimated to have occurred [1]. Non-small cell lung cancer (NSCLC) accounts for 80% of all lung cancers, and approximately 30-40% of patients with NSCLC present with unresectable, locally advanced disease. Concurrent chemoradiation was established as the standard of care for patients with inoperable non-small cell lung cancer (NSCLC) by several randomized trials performed in the 1990s that demonstrated an overall survival benefit with the delivery of concurrent as compared to sequential chemoradiation [2–4]. Despite the survival benefit gained from concurrent chemoradiation therapy, clinical outcomes, particularly locoregional control, remain suboptimal. Previous studies have demonstrated local control rates of 50-70%, but some have reported locoregional control rates as low as 15% [2, 3, 5, 6]. Achieving local control of disease in NSCLC is important, as a recent meta-analysis demonstrated local control significantly improves overall survival results [5].

The use of positron emission tomography/computed tomography (PET/CT) with 2-[^18^ F] fluoro-2-deoxy-D-glucose (FDG) for staging patients with NSCLC has increased over the past two decades and is now considered standard of care in the diagnostic workup in patients with newly diagnosed NSCLC. PET/CT has been shown to have better sensitivity in detecting metastatic disease and to significantly reduce the size of radiation treatment volumes [7–9]. Another potential advantage of PET/CT with FDG is the ability to quantitatively analyze results by using the standardized uptake value (SUV). The SUV of the primary tumor has been shown to correlate with prognosis in NSCLC and may be a marker of disease activity [10, 11]. The predictive value of PET/CT is still undefined, however PET/CT presents an opportunity to develop imaging biomarkers to predict locoregional failure (LRF) and thus refine delivery of radiotherapy in patients with unresectable, non-metastatic NSCLC.

The present study analyzed LRF relative to radiation treatment fields and radiation dose received, in an effort to understand the adequacy of planning treatment volumes and to define anatomic regions most at risk for LRF. Additionally, the pretreatment SUVmax of regions of LRF were analyzed to determine if these regions had a higher SUVmax relative to anatomic regions without LRF.

## Methods

An Emory University Institutional Review Board approved study was performed on newly diagnosed, stage III NSCLC patients undergoing curative intent radiotherapy between 2006 and 2011. ICD-9 codes were used to identify patients with lung cancer treated with radiotherapy between 2006 and 2011. Inclusion criteria included patients with AJCC (7^th^ edition) Stage IIIA and Stage IIIB, newly diagnosed, histologically confirmed NSCLC treated with curative intent radiotherapy. Patients included in this study were treated at Emory University Hospital or Emory University Hospital Midtown. Patients with disease other than stage III were excluded, as were stage III patients treated with surgery and postoperative radiation or patients treated with preoperative chemoradiation. Patients without pre-treatment PET/CT imaging or post-treatment (PET/CT or chest CT) imaging at Emory University were excluded from this study.

Clinical characteristics of patients were extracted from the electronic medical chart, including age, sex, race, histology, T stage, N stage, and administration of chemotherapy. Details of radiation treatment, including dose, treatment technique, and use of daily on-board imaging were obtained through the radiation oncology electronic chart as well as radiation oncology treatment planning software.

### Defining and analyzing locoregional tumor failures

Locoregional tumor failure was defined as recurrent or persistent disease involving the ipsilateral lung, hilum, or mediastinum. Radiology reports and radiology examinations including CT and/or PET/CT were reviewed to determine LRF. Biopsy was not required for confirmation of LRF if serial imaging confirmed persistent or recurrent disease. Supraclavicular recurrences and contralateral lung recurrences were scored as distant failures. Locoregional failures were included as either a first failure event or coincident with distant failure.

For patients who developed a LRF, the CT simulation scan with radiation dose distribution was registered to either the post-treatment CT or PET/CT that documented a LRF. A commercial deformable image registration and dose mapping software (VelocityAI, Velocity Medical Solutions, Atlanta, GA), was used to register the two image studies and extract both dose and SUVmax from the region of interest. The deformable registration algorithm uses free-form deformations based on cubic spline interpolation between sparse, uniformly distributed control points as its transformation model and has been previously described in detail [12–14]. The anatomic region of LRF within the lung, mediastinum, or hilum was contoured, using characteristics including soft-tissue fullness, contrast enhancement, asymmetry and FDG avidity to distinguish tumor from normal, adjacent tissue. Recurrences involving lung parenchyma were contoured on a lung window and recurrences involving soft tissue were contoured on a soft tissue window. A dose volume histogram was created to determine the dose to 95% (D95) of the volume of LRF. Locoregional failures were determined to be in-field (within the 95% isodose line), out-of-field (greater than 2 cm outside the 95% isodose line), or marginal (within 2 cm of the 95% isodose line) based on the relation of the LRF region to the anatomic region encompassed by the radiation fields.

### Integrating Pre-treatment PET/CT

The pre-treatment PET/CT was used to determine the pre-treatment SUVmax of the anatomic region of LRF. SUVmax was defined as the maximum SUV to a voxel location contained within the primary tumor and the involved lymph nodes. The region of LRF was contoured, using the methods described above, on the CT scan documenting LRF. Using the previously described deformable image registration software (VelocityAI), the post-treatment CT scan was registered to the pre-treatment PET/CT scan, and the pre-treatment SUVmax was extracted in the region of LRF. A single user (R.R) performed all image registrations, contouring of LRF, and SUVmax determination.

The SUVmax was quantitatively used to determine FDG-PET activity. SUV was defined as normalizing the tumor uptake of FDG to injected dose per body weight of the patient. SUVmax was defined as the maximum SUV value at a pixel in the volume of interest and was calculated by the image registration software (VelocityAI) from the absolute activity (Bq/mL) in each voxel.

Descriptive statistics were used to illustrate the patient population. Student *t*- tests were used to compare median pre-treatment SUVmax values for patients with LRF versus those patients without LRF, and to compare pre-treatment SUVmax values in anatomic regions of LRF relative to pre-treatment SUVmax values in patients without LRF.

## Results

### Patient characteristics

Sixty-one patients met inclusion criteria for this study. Patient and treatment characteristics are summarized in Table 1. The median age at diagnoses was 65 (range 40–84). Median follow-up time was 19.1 months (range 2.4-76.3 months). The majority of patients (64%) presented with Stage IIIB disease. The most common histology was adenocarcinoma, consisting of 49% of cases. Concurrent chemotherapy was administered in 90% of patients. A platinum-based doublet regimen was administered to all patients receiving concurrent chemotherapy. Carboplatin was given with paclitaxel (42 patients, 76%), pemetrexed (4 patients, 7%), and etoposide (1 patient, 2%). Cisplatin was given with etoposide (6 patients, 11%), pemetrexed (1 patient, 2%), and docetaxel (1 patient, 2%). Six patients received radiotherapy alone. The median radiation dose was 66 Gy (range 39.6-74 Gy). The median pretreatment SUVmax of the primary tumor and involved lymph nodes for the entire patient cohort was 15.2 (range 6.6-50.1).

**Table 1:** **Patient and treatment characteristics**

Characteristics	Patients, n = 61
Median age (range)	65 (40–84)
Sex	
Male	29 (48%)
Female	32 (53%)
Race	
White	39 (64%)
Black	21 (34%)
Other	1 (2%)
Histology	
Adenocarcinoma	30 (49%)
Squamous	18 (30%)
NSCLC NOS*	13 (21%)
T Stage	
T1	9 (15%)
T2	20 (33%)
T3	11 (18%)
T4	19 (31%)
Tx	2 (3%)
N Stage	
N0	1 (2%)
N2	32 (52%)
N3	28 (46%)
AJCC Stage (V.7)	
IIIA	22 (36%)
IIIB	39 (64%)
Radiation Technique	
3-D Conformal	45 (74%)
IMRT	9 (15%)
3-D Conformal & IMRT	7 (11%)
Radiation Dose, median (range)	66 Gy (39.6-74 Gy)
Treatment Planning Margins, median (range)	
GTV to CTV expansion	0.5 cm (0–1 cm)
CTV to PTV expansion	1 cm (0.3-2.5 cm)
Use of daily On-board Imaging (OBI)	24 (39%)
Concurrent Chemotherapy	55 (90%)

*NSCLC NOS- non-small cell carcinoma not otherwise specified.

### Treatment characteristics

All patients underwent CT-based radiation planning. All patients underwent a pretreatment PET/CT that was used to define radiation treatment volumes. Seventy-four percent of patients were treated with 3-D conformal radiotherapy (3DCRT), 15% were treated with IMRT, and 11% were treated with both 3DCRT and IMRT. Of the 45 patients treated with 3DCRT, 35 (78%) patients were treated with anterior-posterior (AP) and posterior-anterior (PA) fields followed by off-cord oblique fields. Seven, 8, or 9-field IMRT was used for the patients treated with IMRT technique. The median gross tumor volume (GTV) to clinical target volume (CTV) margin used was 0.5 cm (range 0–1.0 cm). The median CTV to planning target volume (PTV) margin used was 1 cm (range 0.3-2.5 cm). Elective nodal radiation of non-metabolically active nodal stations was generally not performed. Heterogeneity corrections for treatment planning were used in 31% of patients. On-board imaging, consisting of KV (kilovoltage) orthogonal films, was used in 39% of patients.

### Locoregional tumor failure analysis

**Figure 1:**
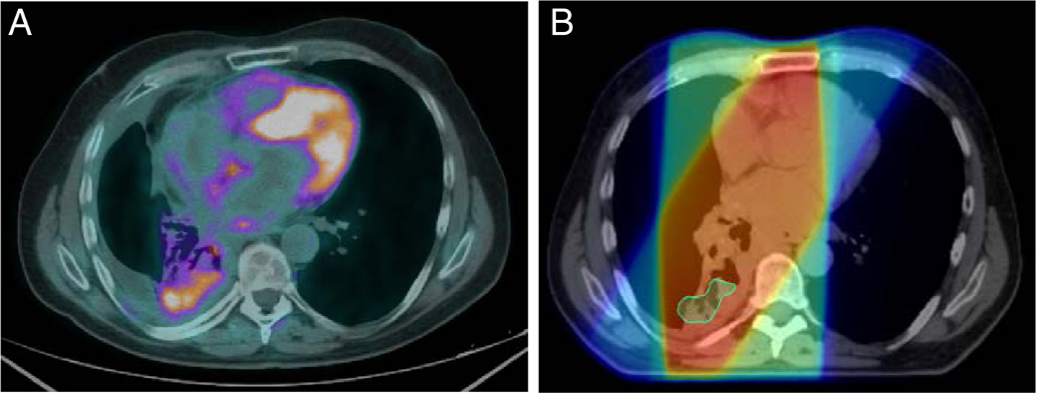
**In-field recurrence. A.)** PET/CT 1 year post-treatment demonstrating recurrence in the posterior right lung. **B.)** Region of PET recurrence contoured and fused to original CT simulation scan. Dose wash on CT simulation reveals the recurrence to be an in-field recurrence with a D95% to the volume of recurrence of 62 Gy.

**Figure 2:**
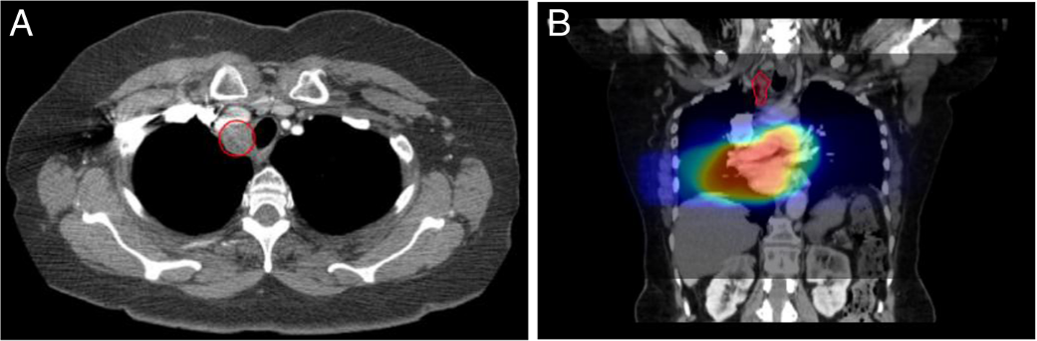
**Out-of-field recurrence. A.)** CT scan 1 year post-treatment demonstrating a recurrence involving a right paratracheal lymph node. **B.)** Region of recurrence contoured and fused to original CT simulation scan. Dose wash on CT simulation scan demonstrates an out-of-field recurrence with a D95% of 1 Gy.

**Figure 3:**
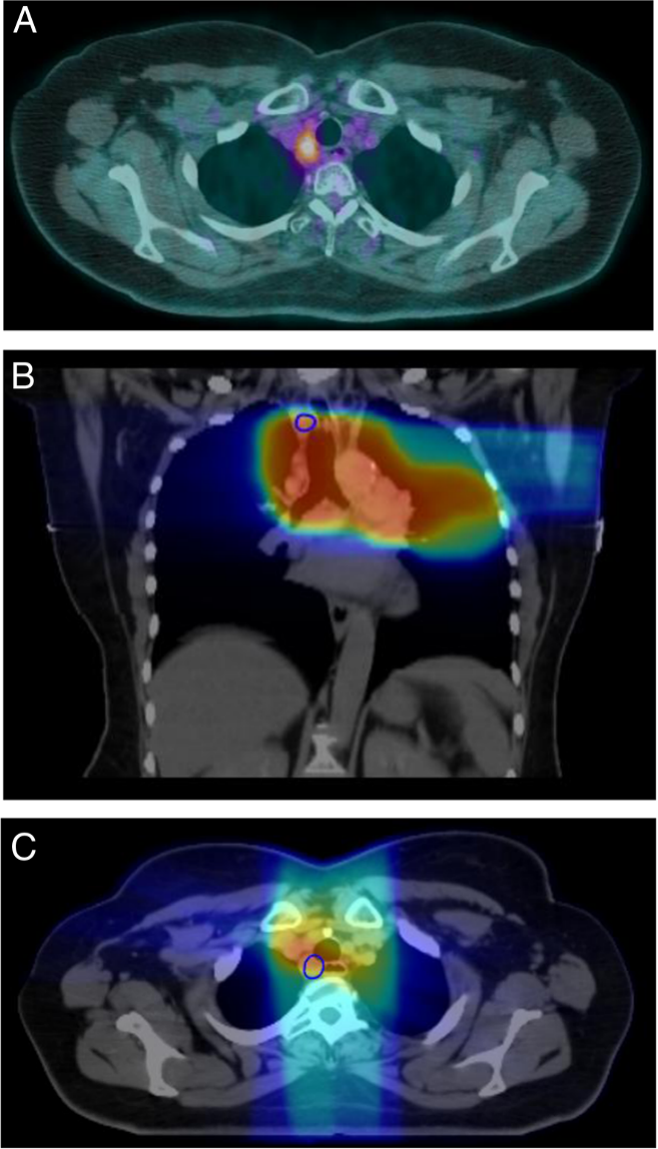
**Marginal recurrence. A.)** PET/CT 4 months post-treatment demonstrating SUV uptake in a right paratracheal lymph node. **B.)** and **C.)** Region of recurrence contoured and fused to original CT simulation scan. Dose wash on CT simulation scan demonstrates a marginal recurrence with a D95% of 50 Gy in a coronal view and axial view, respectively.

Twenty-two patients developed a LRF. The median time to a LRF was 11.4 months (range 3.5-44.6 months). The median radiation dose for patients that developed a LRF was 66 Gy (range 45–66.6 Gy). The median pre-treatment SUVmax of the primary tumor and lymph nodes was 14.9 (range 7.9-50.1) in patients with LRF, but was not significantly different compared with the median pre-treatment SUVmax (15.8, range 6.6-38.6) of patients without LRFs. Five patients developed isolated LRF whereas 17 patients developed LRF and distant failure. Of the 22 patients who developed a LRF, 8 patients developed isolated in-field failures, 6 patients developed isolated out-of-field failures, 4 patients developed in-field and out-of-field failures, and 4 patients developed in-field and marginal failures (Table 2, Figures 1, 2 and 3). No patient developed a marginal failure alone. In total, 73% of patients with a LRF had an in-field component of recurrence. Isolated out-of-field failures occurred in 27% of patients that developed a LRF. Of the out-field recurrences, three patients developed out-of-field nodal recurrences, two patients developed out-of-field lung recurrences, and one patient developed both an elective nodal failure and out of field lung recurrence. Thus, LRF in elective nodal regions occurred in 7% of the entire patient cohort.

**Table 2:** **Patient specific local regional failures in relation to radiation treatment fields**

Location of local regional failure	n = 22 (36%)
In-Field	8 (36%)
In-field and Marginal	4 (18%)
Out-of-field	6 (27%)
In-field and Out-of-field	4 (18%)
Marginal (isolated)	0 (0%)

A total of 39 anatomic regions of failure were identified in the 22 patients that developed a LRF. Fifteen (38%) failures occurred within the parenchyma of the lung and 62% occurred in regional lymph nodes. A total of 22 (56%) anatomic regions of failures occurred in field, 13 failures occurred out of field, and 4 failures occurred in the field edge (Table 3). The median pre-treatment SUVmax for anatomic regions with an in-field failure was 13.4 (range 4.8-43) and was not statistically different from the median SUVmax of patients without a LRF (15.8, range 6.6-38.6). The median D95 to the anatomic region that failed in-field was 63.4 Gy (range 21.3-69.5 Gy). Of the 22 anatomic regions of in-field failure, 12 failures (55%) occurred in the treated mediastinum and 10 failures occurred within the treated lung parenchyma. In the 8 patients with isolated in-field LRFs, a total of 11 anatomic regions were identified; 6 failures occurred within the treated mediastinum and 5 within the treated lung parenchyma. In the 6 patients with isolated out-of-field LRFs, a total of 8 anatomic regions were identified; 5 within the untreated mediastinum, and 3 within the untreated lung parenchyma. Isolated out-of-field LRFs accounted for 21% of all anatomic regions of failure.

**Table 3:** **All cumulative sites of local regional failure**

Location of local regional failure	n = 39
In-Field	22 (56%)
Out-of-field	13 (33%)
Marginal	4 (10%)

## Discussion

Locoregional tumor failure after definitive chemoradiation for Stage III NSCLC remains high despite contemporary radiation techniques and the addition of concurrent chemotherapy. A 36% crude LRF rate was found in this study, which is comparable to previous reports [2, 3, 5]. Interestingly, in this study, despite the use of PET/CT-based planning and a median radiation dose of 66 Gy, LRF after definitive radiation is primarily an in-field event (73%). The marginal failure rate in this study is low, indicating that the GTV to CTV and CTV to PTV margin expansions used in this study are likely appropriate to account for microscopic extent of disease and treatment setup error and tumor motion, respectively.

In-field LRF was not due to inadequate dose, as the median D95 to the anatomic region of LRF was 63 Gy, indicating the region of LRF did receive a tumoricidal dose of radiation. This median dose to regions of LRF is similar to that mandated to the PTV in clinical trials, which typically require 95% of the PTV to receive 100% of the prescription dose. Thus, these anatomic regions where in-field LRF is occurring likely represent radioresistant disease. Strategies to overcome radiation resistance continue to evolve. The Radiation Therapy Oncology Group (RTOG) is currently examining dose escalation to persistently metabolically active disease defined by in-treatment FDG PET (RTOG 11–06). Other strategies under investigation include the addition of molecular agents to standard chemoradiation that target radioresistant pathways.

Curing patients with locally advanced NSCLC is not possible without locoregional disease control. Improving local control has been shown to impact overall survival as demonstrated by a recent meta-analysis comparing concurrent chemoradiation versus sequential chemotherapy and radiation [5]. Phase I and II studies evaluating radiation dose escalation to 74 Gy revealed promising results with significant improvements in progression free survival and overall survival [15–18]. Because of these early promising results, RTOG 06–17 studied the question of dose escalation in a phase III setting. The final results regarding the question of dose were recently presented in abstract form, which revealed a detriment to dose escalation in terms of locoregional failure and overall survival [19]. The LRF rate at 18 months was 35.3% for the 60 Gy arm and 44% for the 74 Gy arm. Based on the results of RTOG 06–17, radiation dose escalation at 2 Gy per fraction does not appear to be the answer to improving local control for patients with Stage III NSCLC, and other strategies are needed to optimize locoregional control.

In this study, 55% of in-field LRFs occurred within the involved mediastinum. Recently, investigators at University of Kentucky reported outcomes of a pilot study evaluating a stereotactic body radiation therapy (SBRT) boost to residual primary lung disease for stage IIA-IIIB NSCLC after 60 Gy of concurrent chemoradiation. Though median follow-up was quite short at 13 months, actuarial local control was 83% [20]. While this approach may be promising, it does not address the mediastinum, which is the more common site of in-field recurrence. At Emory, a phase I dose escalation study to determine the maximum tolerated dose of a SBRT boost to residual primary lung and mediastinal disease after concurrent chemoradiation to a dose of 44 Gy with standard fractionation is currently ongoing.

The use of FDG PET/CT for patients with NSCLC has dramatically increased over the past two decades. Increasing SUVmax of the primary tumor has been associated with a reduction in disease-free survival and overall survival in patients with early stage NSCLC treated with SBRT [21]. FDG PET will likely yield to the development of imaging biomarkers that can aid in predicting LRF in the locally advanced NSCLC population. Determining if pre-treatment SUVmax or other pre-treatment FDG PET parameters are associated with LRF after definitive chemoradiation for Stage III NSCLC is intriguing as this could lead to tailoring of radiation dose escalation to areas thought to be at higher risk for failure. In this study, the median pre-treatment SUVmax for anatomic regions of LRF was 13.4. This SUVmax was not statistically different from the SUVmax of patients who did not develop a LRF. While pre-treatment SUVmax as a potential marker for regions at risk of LRF may not be useful, other PET parameters are currently being explored, including in-treatment FDG/PET CT in the ongoing RTOG 11–06 protocol.

## Conclusions

This study demonstrates that in-field recurrence, particularly nodal recurrence, is the primary mode of LRF after definitive radiotherapy for locally advanced NSCLC. Marginal failures are rare and in-field recurrences are receiving the intended radiation dose. Intrinsic radioresistance is likely contributing to persistent LRF despite improvements in radiation techniques. Pretreatment SUVmax was not associated with LRF in this study, but the use of other PET parameters to determine high-risk regions for LRF remains worthy of further investigation.

## Authors’ information

R.S.R: Resident Physician, Department of Radiation Oncology, Emory University

E.S.: Assistant Professor, Medical Physics, Department of Radiation Oncology, Emory University

T.F.: Director of Medical Physics, Department of Radiation Oncology, Emory University

L.J.S.: Assistant Professor, Department of Radiation Oncology, Emory University

J.J.B.: Professor, Department of Radiation Oncology, Emory University

W.J.C.: Professor and Chair, Department of Radiation Oncology, Emory University

K.A.H.: Assistant Professor, Department of Radiation Oncology, Emory University

## Abbreviations

LRF: Locoregional failure
NSCLC: Non-small cell lung cancer
CT: Computed tomography
PET: Positron emission Tomography
SUV: Standardized uptake value
D95: Dose to 95% of the volume
3DCRT: 3-dimensional conformal radiation therapy
IMRT: Intensity modulated radiation therapy
FDG: Fluorodeoxyglucose
AP: Anteroposterior
PA: Posteroanterior
GTV: Gross tumor volume
CTV: Clinical target volume
PTV: Planning target volume
KV: Kilovoltage
SBRT: Stereotactic body radiation therapy
RTOG: Radiation Therapy Oncology Group.
